# Skeletal Markers of Physiological Stress as Indicators of Structural Violence: A Comparative Study between the Deceased Migrants of the Mediterranean Sea and the CAL Milano Cemetery Skeletal Collection

**DOI:** 10.3390/biology12020335

**Published:** 2023-02-20

**Authors:** Lucie Biehler-Gomez, Andrea Palamenghi, Marie Baudu, Giulia Caccia, Giuseppe Lanza Attisano, Daniele Gibelli, Debora Mazzarelli, Cristina Cattaneo

**Affiliations:** 1 LABANOF, Laboratorio di Antropologia e Odontologia Forense, Sezione di Medicina Legale, Dipartimento di Scienze Biomediche per la Salute, Università degli Studi di Milano, 20122 Milan, Italy; 2 LAFAS, Laboratorio di Anatomia Funzionale dell’Apparato Stomatognatico, Dipartimento di Scienze Biomediche per la Salute, Università degli Studi di Milano, 20133 Milan, Italy

**Keywords:** structural violence, physiological stress markers, migrants, forensic anthropology, cribra orbitalia, porotic hyperostosis, linear enamel hypoplasia

## Abstract

**Simple Summary:**

Although violence is generally understood in its direct form (as physical violence), this is not its only manifestation. Indirect or structural violence results from unequal access to resources and leads to poor quality of health, which may be visible on skeletal remains through various markers. In this paper, three of these markers which may reflect structural violence were examined on skeletal samples of migrants who died during the crossing of the Mediterranean Sea and Italian-born individuals from the CAL Milano Cemetery Skeletal Collection. Results showed that these stress markers were more common and more severe among migrants than non-migrants. The analysis and recognition of such markers in forensic contexts can improve the biological profile by considering the biological effects of cultural, political, and social living conditions.

**Abstract:**

Structural violence is an indirect form of violence that can lead to physiological consequences. Interestingly, these physiological disruptions may affect the skeletons and can therefore provide relevant information on violence and way of life in the analysis of skeletal remains. The aim of the present study was to test the hypothesis that migrants who died in the Mediterranean Sea would present physiological cranial stress markers such as *cribra orbitalia* (CO), porotic hyperostosis (PH), and linear enamel hypoplasia (LEH) more frequently and more severely than Italians of the 20th century. With this intent, a total of 164 crania were examined: 139 from deceased migrants recovered from a shipwreck in the Mediterranean Sea in 2015, aged between 16 and 35 years old, and 25 of the same age from the CAL Milano Cemetery Skeletal Collection. Both presence and severity of CO, PH, and LEH were evaluated. The data obtained were analyzed using Wilcoxon signed-rank and independence Chi-squared tests to compare the results between the two samples and to test whether there was an association between the sample of migrants and the occurrence of lesions. As a result, CO and PH appeared more frequently and more severely in the migrant sample. In addition, migrants were significantly associated with CO, PH, and LEH (*p*-values < 0.05). Although this does not imply in any way that CO, PH, and LEH are specific to migration, they should be regarded as indicators of structural violence.

## 1. Introduction

Violence is a subject of considerable interest in bioarchaeology and forensic anthropology which may bear implications regarding cause of death, the life history of the individual under study, and social dynamics. Yet, violence does not systematically appear in its direct form, that is, manifesting as physical corporeal injuries [1]. Structural violence is an indirect form of violence which consists in the harm inflicted by political, social, and economic structures that provide unequal opportunities for resources (from nutrition to health care) and disparities in power leading to unequal life chances [1]. This differential control over resources as well as their access is responsible for poverty and social inequality which can generate physiological consequences in the form of poor health, malnutrition, and injury [2,3]. This preventable suffering may therefore be embodied among skeletal remains, manifesting as skeletal responses to physiological stressors during growth [3,4,5,6]. Although originating from the disciplines of physics and mechanics, today “stress” is defined as “any type of change that causes physical, emotional or psychological strain” [7]. The term is commonly employed to refer to the feeling of being overwhelmed, under pressure and struggling to cope. In bioarchaeology, “physiological stress” refers to a deviation from physiological homeostatic state and is consequently used as a reliable proxy for estimating some aspects of past health [8,9]. Subsequently, “stress markers” are biological response of the body to a physiological disruption [10].

In the cranium, the most commonly used indicators of such stress are porotic lesions (cribra orbitalia and porotic hyperostosis) and enamel hypoplasia [11,12]. Cribra orbitalia (CO) and porotic hyperostosis (PH) are cribriotic lesions found in the orbital roof and cranial vault, respectively. Traditionally associated with iron-deficiency anemia, their etiology is still debated in the scientific community [13,14,15] and most agree on distinct causes for both lesions [15,16]. However, various conditions may be responsible for porous lesions in the cranium, including scurvy, rickets, hemorrhage, infectious diseases, traumatic injury, some types of tumors [16,17], and even respiratory infections [18]. Some have even suggested that CO may, in fact, correspond to a normal anatomical variant [19]. Given the high frequency of these porous lesions in the bioarchaeological record and the biological mechanics of their development, anemia (megaloblastic, hemolytic and/or iron-deficiency) is considered to be the most probable cause [20]. Chronic anemia can lead to a state of hypoxia. In response to a functional need, the kidneys release erythropoietin hormone to increase red blood cells production and accelerate cellular maturation. This compensatory mechanism stimulates hematopoietic marrow expansion. This expansion of the red marrow, and in particular in the cranium from within the diploë, leads to pressure atrophy of the trabecular and cortical bone, manifesting as an osteoclastic resorption of the trabeculae as well as thinning and perforation of the outer table, rendering visible the porous morphology of the expanding marrow [16,21]. Recent research suggested that CO and PH are generated by different types of anemia: whereas some cases of CO may be caused by marrow hypoplasia and hypocellularity from anemia related to chronic diseases, aplastic/endocrine anemias and renal failure, PH results from marrow hyperplasia in megaloblastic and hemolytic anemias [15,21]. Regardless of their etiology, CO and PH are therefore most commonly indicators of malnutrition or infectious disease during growth [22]. Teeth may also provide information on the events of a person’s life, in particular during body growth. Dental enamel defects, such as linear enamel hypoplasia (LEH), are deficiencies in enamel matrix composition from physiological disruptions [23]. Specifically, they are quantitative defects characterized by deficiencies in the amount or thickness of the enamel, manifesting as macroscopically visible pits or horizontal grooves, caused by an interruption of the amelogenesis from metabolic stress, genetic and epigenetic factors, and resulting in a pathologically thinner enamel [10,24]. Amelogenesis is divided into two stages: secretion and maturation. The former corresponds to the growth of the organic matrix, consisting of amelogenin and non-amelogenin proteins (enameling, amelblastin, tuftelin) [25]. The latter consists in the replacement of the organic matrix with hydroxyapatite crystals, terminating with the apoptosis of ameloblasts [26]. Any disturbance during this process could provoke LEH: deficiencies in calcium, phosphate, vitamins A, C, D, and even variations in pH could interrupt or alter the secretion of hydroxyapatite [26,27,28]. Although most commonly due to nutritional deprivation [22], this disruption of amelogenesis may be due to various stressors including hereditary anomalies, localized trauma, and childhood infectious illnesses [23,24]. In fact, “virtually any environmental factor leading to metabolic disturbance will result in visible changes in the structure of enamel”, as ameloblasts are particularly sensitive to even minor physiological disturbances [20]. Classically seen on the labial surfaces of canines and incisors (although they may be observed on all teeth), they are favored indicators of stress by bioarchaeologists as they develop in tooth crowns during dental growth but do not heal and remodel as CO and PH might, becoming permanent record of childhood stress into adulthood [23].

This interest in applying stress markers as indicators of structural violence to the remains of migrants is relatively new [e.g., [11,29]]. In 2016, Beatrice and Soler suggested that CO, PH, and LEH should be considered a component of the biocultural profile and could be used to distinguish undocumented border crossers (from Mexico and Central America) from American-born individuals. In 2021, they furthered their investigation on the concept of structural violence experienced by undocumented migrants.

Since the early 2010s, Southern European countries are facing a migration crisis that followed social and political uprisings in African and Middle Eastern countries, where thousands of people either chose or were forced to flee their home countries. The Mediterranean Sea is one of the most frequented routes but also the deadliest: attempts at crossing this fictional border have often resulted in mass fatalities; so much so that over 24,000 migrants are estimated to be missing since 2014 (https://missingmigrants.iom.int/region/mediterranean (accessed on January 20 2023)). The Laboratory of Forensic Anthropology and Odontology (LABANOF) is currently involved in the analysis and study of over 300 skulls from migrants who perished in shipwrecks in the Mediterranean between 2013 and 2017, for the construction of biological profiles, and ultimately, for the identification of the victims [30,31].

The aim of the present study is to analyze and compare the presence and severity of three widely recognized cranial stress markers (CO, PH, and LEH) between deceased migrants in the Mediterranean and skeletons of the CAL Milano Cemetery Skeletal Collection [32], representing Italian nationals who died in the 20th century. Given the difficult living conditions in the countries migrants likely originated, our initial hypothesis was that the sample of migrants would exhibit more cranial stress markers and in more severe manifestations than the Italians of the 20th century. This study aims to test this hypothesis and explore its consequences for anthropological practice.

## 2. Materials and Methods

To test the hypothesis that migrants deceased in the Mediterranean Sea present physiological cranial stress markers such as CO, PH, and LEH, more frequently and more severely than Italians of the 20th century, the skulls of a total of 164 individuals were examined (Table 1).

### 2.1. Skeletal Samples

One hundred and thirty-nine skulls constituted the migrant sample, recovered from the shipwreck of 18 April 2015 in the Mediterranean Sea and housed at the Laboratory of Forensic Anthropology and Odontology (LABANOF) for identification purposes. They are currently being examined from a genetic and anthropological perspective for the best possible biological profile to be completed for comparison with future antemortem data [33]. Migratory status of these individuals is demonstrated by the context of retrieval. Among the remains in various stages of decomposition, many skeletonized remains were found commingled; the skulls selected are part of these commingled remains [31]. Sometimes, the crania were found anatomically connected to their respective mandibles. While sex estimations relied upon standard morphological features [34], age-at-death was estimated considering skeletal methods, with the spheno occipital and palatal sutures [35], and dental methods based on the state of eruption and development, translucency of the tooth root and periodontosis, and dental pulp regression, observed macroscopically or through X-rays [36,37,38,39,40,41]. Further details are currently in the process of publication in institutional and scientific reports. Population affinity was performed based on morphological features of the cranium [42]. This method gives probabilities associated to four macro-categories of ancestry: African, American Indian, Asian, and European. The results showed that all crania belonged to male individuals aged between 16 and 35 years, and mainly of African origin [33]. It is important to mention that their “migrant” status was not estimated but already known from the context of the shipwreck. While their exact reason for migrating is not known, “there is no doubt that any person making the decision to cross the Mediterranean at the risk of his or her life has imperative reasons to do so” [43].

Twenty-five skulls were selected from the CAL Milano Cemetery Skeletal Collection to serve as control sample. This skeletal collection, started in 2012, is composed of unclaimed remains buried in Milanese cemetery and housed at the LABANOF [32]. In addition to being contemporary (with individuals who died in the 20th century, including 80% after 1980), the collection has the advantage of being documented, meaning that the individuals are associated to a documentation that includes sex, age-at-death, date of birth, date of death, cause of death, and pathological conditions related to it. The individuals of the CAL Milano Cemetery Skeletal Collection were primarily selected based on their ages-at-death, in order to correspond to the same age range as that of the migrants, that is, between 20 and 35 years. From this criterion, a total of 28 individuals were found in the collection. However, this young age-at-death requirement created a selective mortality bias in the sample which was considered. The non-migrant sample was therefore clearly contextualized and the limitations in inferences were clearly set in order to deal with this selectivity bias. The second selection criterion was the good preservation of the remains, specifically of the cranium and mandible; only skeletons for which the three stress markers selected could be potentially observable (i.e., preservation of the orbits and cranial vault, presence of at least half of the dentition) were selected, which resulted in the removal of three cases from the sample. These two criteria of selection (age-at-death and preservation) thus resulted in a sample constituted of 19 males and 6 females, all of Italian descent. Biological profile was only estimated for the migrant sample, since this data was already known for the cemetery sample.

### 2.2. Analysis of Stress Markers

Each individual in the migrant and non-migrant samples was examined macroscopically for the presence of LEH, CO, and PH.

All observable teeth were examined for LEH and were evaluated as present when enamel anomalies manifested as linear grooves or furrows, were visible to the naked eye and could be felt with a fingernail. LEH was scored as present when at least one tooth showed at least one hypoplastic line.

PH and CO were evaluated as present when porotic lesions could be seen macroscopically on the ectocranial surface of the cranial vault and orbital roofs, respectively. The lesions were then scored for severity according to Stuart-Macadam [44]. Severity scores were collected on all cases of the non-migrant sample and a restricted subsample of 25 skulls of migrants (selected randomly), in order to obtain comparable sample sizes.

### 2.3. Statistical Analyses

A statistical study was realized to compare the presence and severity of the stress markers in both samples. Shapiro–Wilk tests were performed with R (own scripts were written) to verify the normality of the samples and decide whether to use parametric or non-parametric tests for their analysis. Regarding the presence of stress markers and their relationship with the sample, Pearson’s Chi-squared tests of independence were performed with R (own scripts were written) to assess the relationship of the variables “CO”, “PH”, and “LEH” to the sample of migrants. 

Among the migrant group, severity of lesions was evaluated on a subsample of 25 skulls. Wilcoxon signed-rank tests, realized on R (own scripts were written), were applied to the scores of severity of CO and PH to compare their values and assess whether there was a significative difference in severity of the lesions between the migrant and non-migrant samples and in which sample the lesions were more severe. Results were considered significant at *p* ≤ 0.05.

## 3. Results

**Figure 1:**
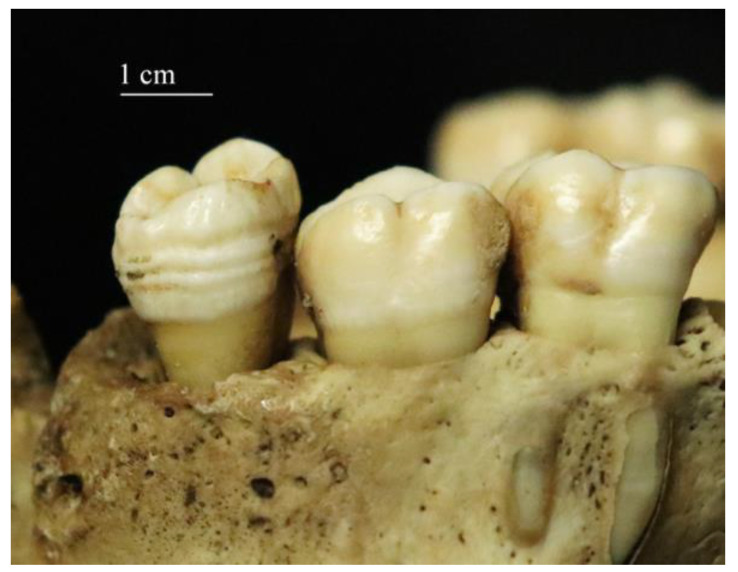
Multiple lines of linear enamel hypoplasia on a maxillary molar of a migrant.

While CO and LEH were present in over half of migrant cases (55%, 76/139), only 24% of the Italian cemetery sample presented such lesions (6/25) (Figure 1). By opposition, PH showed a higher frequency in the non-migrant sample, with 96% of individuals affected (24/25), with respect to the full migrant sample (45%, 63/139) (Table 2 and Table S1).

**Figure 2:**
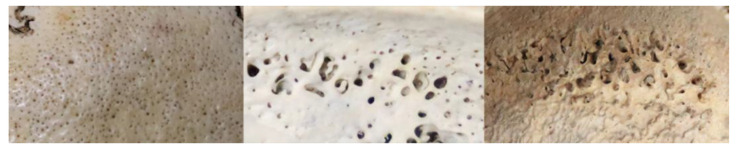
Different severity scores of porotic lesions (left: score 1, middle: score 2, right: score 3).

Scores of severity were applied in the restricted migrant sample of 25 individuals and in the non-migrant sample (Figure 2). In general, orbital lesions in the non-migrant sample were absent (76% of individuals) or coded with low severity (score 1, 83% of the affected sample)—only one individual showed a score 2—whereas all scores were represented in the migrant sample, from light (score 1, 50% of affected individuals—36% of the sample) to severe lesions (score 3, 11% of affected individuals—8% of the sample). PH scores were more varied, with an overall lower severity in the non-migrant sample (score 1, 67% of affected individuals) with respect to the migrant sample (scores 2 and 3, 80% of individuals) (Table 3).

These observations on severity scores were confirmed by statistical analyses which showed: first, that non-parametric tests should be run based on tests of normality (*p*-values < 0.05); and second, that the reduced migrant sample presented more severe CO and PH lesions with respect to the non-migrant samples (*p*-values < 0.05) (Table 4).

In addition, when considering the entirety of the sample (i.e., 164 skulls), independence Chi-squared tests showed that the variable “migrant” presented a statistically significant association with “CO”, “PH”, and “LEH” (*p*-values < 0.05). However, when testing the association of the variables “migrant” and “CO and/or PH and/or LEH” the result was not statistically significant (*p*-value > 0.05).

## 4. Discussion

The results of the evaluation of stress markers on the population of migrants showed high frequencies of LEH, PH, and CO. In the “reduced” migrant sample of 25 individuals, where severity of porotic lesions were scored, all expressions of the lesions were found, ranging from “scattered fine foramina” (score 1) to “outgrowth in trabecular structure from the normal contour of the outer bone table” (score 3) [44]. Alternatively, CO and LEH were uncommon in the non-migrant sample, absent in over three quarters of individuals, and the severity of CO was very low (83% of individuals affected coded score 1). Although PH was more frequent in this sample, found in 96% of the individuals studied, it showed a low severity with 67% of individuals affected with a score 1 (Table 2 and Table S1).

It is important to specify that while mandibles were observable for all individuals selected from the CAL Milano Cemetery Skeletal Collection, only 33% (46/139) of migrants had a complete skull (i.e., cranium + mandible). In the non-migrant sample, four individuals presented LEH (16%), and in three of them (75% of the affected individuals), mandibular teeth presented hypoplastic defects. In the migrant sample, 76 individuals had LEH but only 46 skulls in total were complete with the mandible. Of the 24 complete skulls with LEH, 17 (or 71%) presented enamel defects on mandibular teeth, which shows the importance of the mandible for the detection of LEH. Consequently, LEH results in the migrant sample actually represent a minimum frequency, as crania without mandible may have presented LEH in mandibular teeth which went undetected in their absence. Antemortem tooth loss was a rare occurrence in the migrant sample, but it was observed in some cases of the non-migrant sample, in particular the four individuals whose associated documentation mentioned a history of narcotics and drug addiction, while postmortem tooth loss had occurred in both samples. Yet at least 50% of the dentition was present in each individual of both samples, allowing for the observation of LEH in the remaining teeth and partially mitigating this selectivity bias. Nonetheless, this bias in the preservation of the teeth may be responsible for loss of information regarding LEH and may have resulted in underestimation of the frequencies of the enamel defect in both samples.

Overall, migrants were significantly more susceptible to present CO, PH, and LEH than non-migrants and in more severe expressions of the lesions (*p* < 0.05). This is consistent with previous studies on stress markers on migrants [11,29]. Indeed, Beatrice and Soler [29] showed that undocumented border crossers at the Mexico/USA border in South Arizona were 7.91 times more likely to exhibit PH and 3.01 times more likely to present LEH than American-born individuals; however, the difference for CO was not significant at *p* < 0.05. The results of the present study further those of Beatrice and Soler [29] as they show CO as significantly more present in migrants of the Mediterranean than Italian-raised individuals and demonstrate that in addition to presence, severity of porotic lesions (both CO and PH) should be considered, as there are significantly more severe in migrants, strengthening the results of Beatrice and Soler [29].

The high frequency and severity of stress markers in the migrant sample may be explained by the living conditions of these individuals in their early life. While country of (declared) nationality is one of the characteristics registered in Europe at the arrival of migrants crossing the Mediterranean, it is important to consider that the data is often incomplete and biased (for instance, they may try to provide answers that they believe will facilitate their asylum claim) [43]. Moreover, migrants often undertake a long route, crossing several borders prior to embarking on the boat across the sea; hence, material documents or money from specific countries that they may be carrying do not constitute reliable indicators of their country of origin. Nevertheless, police data count about 70% of migrant arrivals by sea to Europe originating from sub-Saharan Africa [43] and our own analyses support an origin of the migrants under study from sub-Saharan countries [33]. In this region of the world, iron deficiency and iron deficiency anemia are still considered public health issues by the World Health Organization, with a prevalence of anemia sometimes exceeding 60% in children [45]. Malnutrition and food insecurity are two other major public threats, especially among children. The UNICEF reported in 2016 that one in three children under 5 years was malnourished in the continent, and that “while consumption was generally satisfactory [144–306% of recommended daily amounts (RDA)], inadequate intakes were found for vitamin A, calcium, zinc, and iron.” [45]. Malnutrition is influenced by several factors, including restricted food access, low income, geographical location, humidity, high exposure to the sun, altitude and latitude, soil and crop quality, and climate change (e.g., droughts and floods) [45,46]. Sub-Saharan Africa registers the highest prevalence of underweight children and infant/child mortality in the world [46]. Moreover, sub-Saharan Africa also counts the most affected countries by malaria (i.e., Nigeria and the Democratic Republic of Congo) which alone register over a third of global cases [45]. The high prevalence of malaria only exacerbates that of existing anemia. Viruses, bacteria, and parasites are also common in children. Parasitic infections often occur in children because of exposure to contaminated water sources in absence of other safer sources, with parasites being the third most common cause of infectious diarrhea in sub-Saharan children [47]. Intestinal parasites cause malnutrition and impairment of physical development in children. Gastroenteritis and HIV/AIDS are other serious health conditions affecting children. It is estimated that by age 5, many children are already infected by hepatitis-B. In addition, pneumonia is a major complication in HIV-infected children and one of the main causes of fever in children under 5 years [47].

Although stress markers were present in smaller percentages (Table 2) in the Italian population, they were still observable. This result may be explained by the composition of the non-migrant sample. Indeed, these individuals were selected from the CAL Milano Cemetery Skeletal Collection based on their age-at-death, in order to be comparable to the migrant sample: as porotic cranial lesions can remodel over time, the samples had to present similar age ranges. This created a selective mortality bias in the sample: the individuals selected died at a relatively young age, between 20 and 35 years, in a country (Italy) and at a time (1944–2000) when life expectancy was higher by far (about 50 years in 1944 to 80 years in 2000 [48]). Additionally, the 1980s and 1990s were marked by the HIV pandemic, which happened to be the cause of death of 16% (4/25) of the individuals in the sample. Although no link has yet been established between HIV and stress markers on skeletal remains [49], this is a factor impacting health that should be acknowledged. Moreover, 8% (2/25) of the individuals had a history of narcotics and drug addiction, which is evidenced in their poor oral health marked by numerous ante-mortem tooth loss, caries, abscesses, and heavy dental wear. Even though we tried to select individuals as contemporary as possible, the years of death had to be expanded to satisfy the criteria of selection (i.e., the young age-at-death, not common in the collection), and so 32% of the individuals died before 1970 (8/25). These individuals, born as early as 1922, lived through different social and economic crises, the Second World War, and its aftereffects which may explain the presence of markers of physiological stress during early life. Consequently, the control sample of Italian individuals is not representative of the current Italian population, and the presence of markers of physiological stress is therefore not surprising.

CO, PH, and LEH are indicators of systemic non-specific physiological stress in the first decades of life, such as nutritional deprivation and infections. The high prevalence of these lesions in the migrant sample indicates that these individuals underwent episodes of physiological disruption during growth. In this context, the presence of these lesions evidences that these early life experiences incorporated into their bodies. Although the exact causes responsible for the events of physiological disruption cannot be identified given the lack of information on their context of origin, these lesions can be related to the poor living conditions and systemic poverty of migrants during their early life. As such, they constitute embodied structural violence because “they are evidence of differential exposure to preventable biological harm caused indirectly by social and economic inequalities” in a global context [11].

However, despite their significantly higher frequency, severity, and association with migrants with respect to the Italian sample, they do not constitute markers of migration. Beatrice and Soler [29] suggested that cranial stress markers should be considered a component of the biological profile which may distinguish migrants from non-migrants. We agree with this inasmuch as these physiological stress markers are signs of embodied suffering and in this case, structural violence, of which we know undocumented border crossers and migrants crossing the Mediterranean at the risk of their life endured more than American-born and European-born individuals, respectively. Nonetheless, the same reasoning may be applied to other demographic groups who suffer from structural violence. CO, PH, and LEH are in no way specific to migration and should be regarded as signs of embodied suffering. As such, we suggest that stress markers should be considered in forensic anthropology as they can provide information on way of life and the less visible side of violence, that is, structural violence.

## 5. Conclusions

A total of 139 migrant and 25 Italian cemetery remains were examined for the presence and severity of CO, PH, and LEH. As a result, this study has shown that migrants present more frequently and severely these stress markers with respect to the Italian sample, and that these cranial lesions are embodied signs of the physiological disruption they experienced. Nonetheless, it is important to emphasize that the presence of these stress markers does not identify migration but represents embodied suffering. As a response to systemic physiological stressors during growth, skeletal remains may manifest bone lesions, such as CO, PH, and LEH. In this context, the poor living conditions and general poverty in which the migrants were living during their early life, indirectly caused by social and economic inequalities, generated preventable biological harm (including malnutrition, dietary insufficiencies and infections), a hallmark of structural violence, which was embodied through these bone lesions. Considering these lesions in forensic anthropology can therefore provide useful information regarding living conditions and gives a new dimension to the study of skeletal remains, furthering anthropological analysis from the construction of a biological profile (limited to biological characteristics) to a biocultural one, which includes the biological effects of cultural and social living conditions. Biological anthropology therefore has the capacity not only to reconstruct the past or help assist identification in contemporary scenarios, but also can provide insight and remind us of current situations of indirect violence and poverty which need to be contrasted.

## Figures and Tables

**Table 1:** Details of the study sample.

Sample	*n*	*n* Males	*n* Females	Age Range
Migrants	139	139	0	18–35
CAL Milano Cemetery Skeletal Collection	25	19	6	20–35 (mean: 27.5, median: 28)

**Table 2:** Frequencies of cribra orbitalia (CO), porotic hyperostosis (PH), and linear enamel hypoplasia (LEH) in the samples.

Sample	*n*	Mandible		CO		PH		LEH	
		*n*	%	*n*	%	*n*	%	*n*	%
Migrant sample	139	46	33%	75	54%	63	45%	76	55%
“Reduced” migrant sample	25	3	4%	17	68%	25	100%	6	24%
Non-migrant sample	25	25	100%	6	24%	24	96%	4	16%

**Table 3:** Severity scores in the “reduced” migrant sample of 25 individuals and non-migrant sample (% refer to percentages of affected individuals).

	Severity Score	Migrant Sample		Non-Migrant Sample	
		*n*	%	*n*	%
Cribra orbitalia	Score 1	9	50%	5	83%
	Score 2	7	39%	1	17%
	Score 3	2	11%	0	0%
Porotic hyperostosis	Score 1	5	20%	16	67%
	Score 2	18	72%	8	33%
	Score 3	2	8%	0	0%

**Table 4:** Statistical analyses and *p*-values (significant *p*-values were bolded in the table).

Variables	Shapiro *p*-Values	Wilcoxon *p*-Values	Chi-Squared *p*-Values
Sample migrants~values CO	**0.00291**		
Sample migrants~values PH	**7.509 × 10^−6^**		
Sample non-migrants~values CO	**3.733 × 10^−7^**		
Sample non-migrants~values PH	**9.42 × 10^−6^**		
Severity CO migrants~non-migrants		**0.00025**	
Severity PH migrants~non-migrants		**0.0002303**	
Samples~CO			**0.009136**
Samples~PH			**8.332 × 10^−6^**
Samples~LEH			**0.0008246**
Samples~CO and/or PH and/or LEH			0.4366

## Data Availability

Data available within the article or its Supplementary Materials.

## Supplementary Materials

The following supporting information can be downloaded at: https://www.mdpi.com/article/10.3390/biology12020335/s1, Table S1: Scoring details on the « reduced » migrant sample of 25 individuals and non-migrant sample (values in red indicate higher or lower values than the mean average at 95% confidence, showing scores 3 in the migrant sample and score 2 for CO in the non-migrant sample as unusually high).
