# Supplementary material for: Skeletal Markers of Physiological Stress as Indicators of Structural Violence: A Comparative Study between the Deceased Migrants of the Mediterranean Sea and the CAL Milano Cemetery Skeletal Collection
Source: Biology (Basel). 2023 Feb 20;12(2):335. doi: 10.3390/biology12020335 (PMC9953607; doi:10.3390/biology12020335)
Supplement: Supplementary file 1 [file biology-12-00335-s001.zip › biology-2220978-supplementary.pdf]

Supplementary Table S1: scoring details on the « reduced » migrant sample of 25 individuals and non-migrant sample (values in red indicate higher or lower values than the mean average at 95% confidence, showing scores 3 in the migrant sample and score 2 for CO in the non-migrant sample as unusually high)

Sample	Individual	Score CO	z-score	Score PH	z-score	n° teeth with LEH (dental notation ISO 3950)
"Reduced " migrant sample	421	-	-	2	0,23	-
	423	-	-	2	0,23	-
	424	2	0,96	2	0,23	-
	427	2	0,96	2	0,23	-
	428	-	-	1	-1,67	-
	431	1	-0,08	2	0,23	-
	432	2	0,96	2	0,23	-
	433 C1	2	0,96	2	0,23	-
	433 C2	-	-	2	0,23	18
	433 C3	1	-0,08	1	-1,67	-
	433 C4	-	-1,13	2	0,23	-
	434	1	-0,08	2	0,23	-
	436	1	-0,08	2	0,23	-
	437	-	-	2	0,23	-
	438 C	-	-	1	-1,67	11-14-17-21-22-23-24-25-27-32-33-35-36-43-44-45-46
	439	2	0,96	2	0,23	11-12-13-21
	441 C1	1	-0,08	2	0,23	-
	441 C2	1	-0,08	3	2,13	-
	442	-	-	2	0,23	-
	451	1	-0,08	2	0,23	-
	454	2	0,96	2	0,23	16-26
	458 C1	1	-0,08	2	0,23	23-24-25
	458 C2	3	2,01	3	2,13	-
	458 C3	1	-0,08	1	-1,67	-
	458 C4	3	2,01	1	-1,67	13
Non-migrant sample	27	-	-	1	-0,52	-
	185	-	-	1	-0,52	-
	347	2	3,13	1	-0,52	-
	409	1	1,31	1	-0,52	-
	434	-	-	1	-0,52	-
	475	-	-	2	1,33	-
	483	-	-	1	-0,52	-
	487	-	-	1	-0,52	-
	541	1	1,31	2	1,33	-
	548	-	-	2	1,33	-

	576	-	-	1	-0,52	-
	774	-	-	1	-0,52	-
	794	1	1,31	1	-0,52	
	855	-	-	-	-	-
	857	-	-	1	-0,52	-
	1213	-	-	1	-0,52	-
	1219	-	-	2	1,33	-
	1251	-	-	2	1,33	-
	1253	1	1,31	2	1,33	-
	1254	-	-	1	-0,52	-
	1255	-	-	1	-0,52	16-26-42
	1259	1	1,31	2	1,33	15-27-33-34-35-41-42-43-47
	1260	-	-	1	-0,52	21
	1261	-	-	2	1,33	-
	1343	-	-	1	-0,52	33-43
